# Cross-cultural adaptation and psychometric validation of the Spanish version of the Orthorexia Nervosa Inventory (ONI-ES)

**DOI:** 10.3389/fnut.2026.1822964

**Published:** 2026-05-20

**Authors:** Esther Hidalgo-Velez, María Dolores Onieva-Zafra, Antonio Hernández-Martínez, Minerva Munera-Jareño, Alberto Bermejo-Cantarero, María Laura Parra-Fernández

**Affiliations:** Department of Nursing, Phisyoterapy and Ocupational Therapy, University of Castilla-La Mancha. Faculty of Nursing, Ciudad Real, Spain

**Keywords:** cross-cultural adaptation, eating behavior, health behavior, orthorexia nervosa, psychometric validation

## Abstract

**Background:**

Orthorexia nervosa (ON) has gained increasing attention as a maladaptive pattern of rigid health-oriented eating characterized by cognitive preoccupation, behavioral restriction, and functional impairment. The Orthorexia Nervosa Inventory (ONI) was developed as a multidimensional instrument to assess orthorexic tendencies; however, further cross-cultural validations are needed to ensure its applicability across different linguistic and cultural contexts.

**Objective:**

To translate, culturally adapt, and evaluate the psychometric properties of the Spanish version of the Orthorexia Nervosa Inventory (ONI-ES) in a general adult population in Spain.

**Methods:**

A two-phase validation study was conducted. Phase 1 involved forward–backward translation and expert panel evaluation to assess content validity using Aiken’s V coefficient. Phase 2 examined construct validity and reliability in two independent samples (EFA: *n* = 272; CFA: *n* = 257; total *N* = 529). Exploratory factor analysis (EFA) was performed using Principal Axis Factoring with oblimin rotation, followed by confirmatory factor analysis (CFA) using maximum likelihood estimation. Internal consistency was assessed using Cronbach’s alpha and McDonald’s *ω*, and temporal stability was evaluated through test–retest reliability using the intraclass correlation coefficient (ICC).

**Results:**

All items demonstrated adequate content validity (Aiken’s V range: 0.71–0.96). EFA supported a three-factor structure—orthorexic behaviors, emotional attitudes toward food, and social/functional impairment—accounting for 41.9% of the total variance. CFA confirmed the adequacy of the three-factor model (CFI = 0.905; TLI = 0.891; RMSEA = 0.066). The ONI-ES showed excellent internal consistency (*α* = 0.91) and good to excellent temporal stability (ICC = 0.824–0.903). Convergent validity was supported by a positive correlation with EAT-26 scores (*r* = 0.586, *p* < 0.001).

**Conclusion:**

The ONI-ES demonstrates solid psychometric properties and replicates the original three-factor structure in a Spanish general population. These findings support its reliability, structural validity, and cross-cultural applicability as a multidimensional measure of orthorexic symptomatology.

## 1 Introduction

Orthorexia nervosa (ON) has gained increasing attention in recent years as a pattern of eating behavior characterized not merely by an interest in healthy eating, but by a rigid and obsessive preoccupation with food quality that progressively dominates daily life (1–4). Unlike normative health-oriented dietary practices, orthorexic behaviors are marked by inflexibility, heightened anxiety in response to perceived dietary transgressions, and a tendency to prioritize dietary rules over social, occupational, or emotional needs (5–7). This distinction between adaptive and maladaptive forms of healthy eating has become particularly relevant in contemporary societies, where wellness discourses and moralized views of food are increasingly normalized (8, 9).

Beyond dietary practices themselves, orthorexic tendencies have been associated with a range of negative psychological and social consequences (10). As rigid food rules intensify, individuals may experience increased emotional distress, including guilt, anxiety, and a heightened sense of self-criticism following perceived dietary deviations. Over time, these patterns can lead to progressive social withdrawal, interference with interpersonal relationships, and reduced participation in everyday activities, suggesting that the core problem lies not only in what is eaten, but in the functional impact of eating-related cognitions and behaviors (11–13). This functional impairment represents a key element in distinguishing orthorexia nervosa from non-pathological health-conscious eating (14).

The absence of formally established diagnostic criteria for orthorexia nervosa has shifted much of the conceptual and empirical work in this field toward its measurement. In the absence of an official diagnostic framework, research has relied predominantly on self-report instruments to operationalize the construct, resulting in considerable variability in how orthorexia nervosa is defined and quantified across studies (15–18). Although expert consensus efforts have sought to clarify core dimensions—such as obsessive preoccupation with food quality and behavioral rigidity—these proposals have not been uniformly translated into assessment tools (19, 20). Consequently, widely used instruments, including the ORTO-15 and related measures, differ substantially in their conceptual scope and psychometric robustness, reflecting diverse operationalizations of orthorexia nervosa across studies (17, 21). This reliance on heterogeneous instruments has contributed to inconsistent prevalence estimates and ongoing debates regarding the boundaries between health-oriented eating and maladaptive orthorexic patterns (22–24).

Within this context, the Orthorexia Nervosa Inventory (ONI) has been proposed as a multidimensional instrument aimed at operationalizing orthorexic tendencies in a more comprehensive manner (25). Beyond its original development, the ONI has been adapted and validated by different research groups across various cultural contexts (26–29). In Italy, the validation conducted in a general adult population confirmed the original three-factor structure, showing satisfactory model fit indices and high internal consistency (26). Similarly, studies carried out in Turkey, including both adolescent and adult clinical samples, reported adequate factorial validity and reliability (27). More recently, the Chinese version of the ONI, developed through a rigorous translation and cross-cultural adaptation process, replicated the three-dimensional structure and provided additional evidence of the instrument’s temporal stability (28). Collectively, these studies suggest that the ONI offers a consistent framework for assessing orthorexic tendencies across different populations, contributing to a clearer differentiation between health-oriented eating behaviors and more problematic orthorexic patterns.

In Spanish-speaking contexts, several instruments have been translated and validated to assess orthorexic tendencies, reflecting the growing interest in this construct (30–33). However, given the conceptual diversity of existing measures and the multidimensional approach underlying the ON, the validation of additional instruments remains relevant to advance research in this field. Extending the cross-cultural validation of the ONI to Spanish populations may contribute to broadening the range of available assessment tools and facilitating comparisons across studies conducted in different cultural settings. Accordingly, the aim of the present study was to translate and culturally adapt the Orthorexia Nervosa Inventory into Spanish (ONI-ES) and to examine its psychometric properties in a general adult population in Spain.

## 2 Methods

### 2.1 Study design

The study was conducted in two sequential phases.

*Phase 1* focused on the translation and cross-cultural adaptation of the Orthorexia Nervosa Inventory into Spanish. This phase included forward translation, synthesis of the translations, back-translation, and expert committee review to assess content validity. Expert judgment was used to evaluate item relevance, clarity, and adequacy, and minor linguistic refinements were implemented when necessary to ensure conceptual, semantic, and cultural equivalence with the original instrument.

*Phase 2* aimed to evaluate the psychometric properties of the Spanish version of the instrument. Construct validity was examined through exploratory and confirmatory factor analyses conducted in two independent samples. Reliability was assessed by analyzing internal consistency and temporal stability using a test–retest procedure. This two-phase approach allowed for a comprehensive evaluation of the ONI-ES, addressing both its cultural adaptation and its psychometric performance in a general adult population.

### 2.2 Ethical approval and consent to participate

The study protocol was approved by the Research Ethics Committee with Medicines of the Integrated Healthcare Area of Ciudad Real (Spain) at its session held on 21 March 2023 (No. 03/2023, approval code C-603). All procedures were conducted in accordance with the ethical principles outlined in the Declaration of Helsinki. Prior to participation, all individuals provided written informed consent, and the confidentiality and anonymity of the collected data were guaranteed.

### 2.3 Participants

Two independent samples were recruited for the psychometric validation process. The first sample consisted of 272 participants and was used for the exploratory factor analysis (EFA), while the second sample included 257 participants and was employed for the confirmatory factor analysis (CFA). The use of independent samples for EFA and CFA was intended to avoid over fitting and to enhance the robustness of the factor structure.

For both samples, a non-probabilistic convenience sampling method was applied. Participants were recruited nationwide through online questionnaires disseminated via social media and digital platforms. Sample sizes were considered adequate to perform exploratory and confirmatory factor analyses, in line with commonly accepted recommendations for psychometric validation studies.

All participants provided written informed consent prior to inclusion, and the collected data were fully anonymized for analysis, ensuring confidentiality and the voluntary nature of participation.

### 2.4 Instruments

#### 2.4.1 Demographic information

Participants provided demographic and lifestyle information through a brief questionnaire developed for the purposes of this study. The collected variables included sex, age, marital status, socioeconomic level, and occupational status. Additional lifestyle and health-related variables were also recorded, including self-reported dietary pattern, time spent walking or cycling as an indicator of physical activity, and body mass index (BMI). Participants were also asked about family history of eating-related conditions, including eating disorders, obesity, anorexia, bulimia, binge eating, unspecified eating disorders, and food-related anxiety. These variables were used to describe the characteristics of the study samples and to examine potential differences between the subsamples used for exploratory and confirmatory analyses.

#### 2.4.2 Orthorexia nervosa inventory (ONI)

The Orthorexia Nervosa Inventory (ONI) is a 24-item multidimensional self-report instrument designed to assess orthorexic symptomatology as a structured pattern of maladaptive health-oriented eating (25). The instrument comprises three theoretically derived dimensions: orthorexic behaviors, emotional preoccupation, and impairment. In the original factorial structure, the factor behaviors is assessed through items 2, 4, 6, 8, 11, 15, 17, 18 and 22; the factor emotions is captured by items 1, 9, 13, 21 and 23; and the factor impairment is evaluated through items 3, 5, 7, 10, 12, 14, 19, 20 and 24. Items are rated on a Likert-type scale, with higher scores reflecting greater orthorexic symptomatology. No items are reverse-scored in the original version of the instrument.

#### 2.4.3 Eating attitudes test (EAT-26)

The Eating Attitudes Test (EAT-26) is a widely used self-report instrument designed to assess symptoms and concerns characteristic of eating disorders (34, 35). Originally derived from the EAT-40, the EAT-26 serves as a screening tool for identifying disordered eating attitudes and behaviors, particularly those associated with anorexia nervosa and related restrictive patterns. The instrument comprises 26 items rated on a Likert-type scale, assessing dieting behaviors, bulimic tendencies, food preoccupation, and oral control. Higher total scores indicate greater levels of eating disorder symptomatology. The EAT-26 has demonstrated satisfactory psychometric properties across diverse cultural and population contexts and is commonly used in both clinical and non-clinical samples. In the present study, the total EAT-26 score was used to examine the convergent validity of the ONI-ES.

### 2.5 Translation and cross-cultural adaptation procedure

#### 2.5.1 Forward translation

The original English version of the Orthorexia Nervosa Inventory (ONI) was independently translated into Spanish by two bilingual translators. One translator was a health professional with experience in eating disorders and familiarity with the construct of orthorexia nervosa, while the second translator was a professional translator with no clinical background and blinded to the study objectives. This approach was intended to ensure both conceptual accuracy and linguistic naturalness.

#### 2.5.2 Synthesis of the translations

The two forward translations were compared and synthesized into a single preliminary Spanish version through consensus among the research team. Discrepancies between the translations were discussed, and decisions were made based on semantic equivalence, conceptual relevance, and cultural appropriateness. This process resulted in a unified Spanish version of the ONI for subsequent phases.

#### 2.5.3 Back-translation

The synthesized Spanish version was independently back-translated into English by two bilingual translators whose mother tongue was English and who were unfamiliar with the original version of the ONI. The back-translated versions were compared with the original instrument to identify potential inconsistencies or deviations in meaning. Any discrepancies were reviewed and resolved by the research team to ensure conceptual equivalence with the original version.

#### 2.5.4 Expert committee review

After obtaining the preliminary Spanish version of the Orthorexia Nervosa Inventory (ONI-ES), the instrument was evaluated by a multidisciplinary panel of experts to assess its content validity. A total of sixteen experts from different professional backgrounds participated in this phase, including five psychiatrists, six psychologists, and five mental health specialist nurses. Experts were selected based on their clinical and research experience in eating disorders, mental health, and psychometric assessment. To ensure a broad range of perspectives, professionals from different geographical regions of Spain were included.

Experts were contacted via email and invited to participate as reviewers of the adapted instrument. After agreeing to participate, they received the questionnaire and were asked to independently evaluate each item using a Likert-type scale ranging from 1 (lowest score) to 5 (highest score). Items were assessed according to four criteria: wording, clarity and comprehensibility, relevance to the construct of orthorexia nervosa, and overall adequacy. In addition, an open-ended section was provided to allow experts to include qualitative comments or suggestions for item refinement.

Item-level content validity was assessed using Aiken’s V coefficient, which is appropriate for ordinal rating scales and expert judgment. Aiken’s V coefficients and their corresponding 95% confidence intervals were calculated for each item to quantify the degree of agreement among experts regarding item relevance and adequacy.

Once all expert evaluations were collected, the results were analyzed and discussed by the research team. Based on the quantitative indices and the qualitative feedback provided by the experts, minor wording modifications were made to improve clarity and semantic precision when necessary, resulting in version 1 of the ONI-ES. No items were eliminated at this stage, as all items demonstrated adequate content validity.

### 2.6 Data collection procedure

Data were collected using an online self-administered questionnaire that included the final Spanish version of the Orthorexia Nervosa Inventory (ONI-ES) and a brief set of sociodemographic questions.

Data collection was designed to obtain two independent samples for the psychometric analyses. One sample was used for the exploratory factor analysis (EFA), and a separate sample was used for the confirmatory factor analysis (CFA). Measures were implemented to ensure that each participant could complete the questionnaire only once and that no individual was included in both samples.

Only fully completed questionnaires were included in the statistical analyses. Prior to analysis, the data were screened to identify incomplete responses or inconsistencies.

### 2.7 Statistical analysis

Descriptive statistics were used to summarize the characteristics of the study samples. Qualitative variables were described using absolute and relative frequencies, while quantitative variables were summarized using means and standard deviations (SD).

Content validity was assessed through expert judgment using Aiken’s V coefficient at the item level, together with its corresponding 95% confidence intervals.

Construct validity was examined through exploratory and confirmatory factor analyses. To avoid capitalization on chance and to enhance the robustness of the factorial solution, the total sample was divided into two independent subsamples. The participant-to-item ratio exceeded 10:1 in both subsamples, satisfying commonly recommended criteria for factor analysis and supporting the adequacy of the sample size for exploratory and confirmatory procedures. Exploratory factor analysis (EFA) was conducted on the first subsample to identify the underlying factorial structure of the Spanish version of the Orthorexia Nervosa Inventory (ONI-ES). Prior to factor extraction, the suitability of the data was evaluated using the Kaiser–Meyer–Olkin (KMO) measure of sampling adequacy and Bartlett’s test of sphericity. Factor extraction was performed using Principal Axis Factoring (PAF), followed by oblimin rotation to allow correlations among factors. PAF was selected as it is more appropriate for identifying latent psychological constructs. The number of factors retained was determined based on eigenvalues greater than 1, inspection of the scree plot, theoretical coherence with the original instrument, and parallel analysis based on simulated random data. The parallel analysis supported the retention of three factors, and no alternative factorial solution demonstrated superior interpretability or conceptual consistency. The resulting factorial structure was subsequently evaluated within a latent variable framework through confirmatory factor analysis (CFA) conducted in an independent sample, thereby strengthening the construct validation process.

Confirmatory factor analysis (CFA) was subsequently performed on the second independent subsample to test the factorial structure identified in the exploratory phase. Model estimation was carried out using the maximum likelihood method. Prior to CFA, assumptions of multivariate normality were examined, and no severe deviations were observed. Model fit was evaluated using the Comparative Fit Index (CFI), the Tucker–Lewis Index (TLI), and the Root Mean Square Error of Approximation (RMSEA). Values of CFI and TLI ≥ 0.90 and RMSEA ≤ 0.08 were considered indicative of acceptable model fit.

Convergent validity was examined by calculating Pearson correlation coefficients between ONI-ES total scores and EAT-26 scores using the full sample.

Internal consistency was assessed using Cronbach’s alpha coefficients for the total scale and each subscale. Item-level analyses were conducted to examine whether the removal of any individual item resulted in an improvement in overall reliability.

Temporal stability was evaluated through a test–retest procedure conducted in a subsample of 31 participants who completed the questionnaire on two occasions separated by a 15-day interval. Test–retest reliability was assessed using the intraclass correlation coefficient (ICC) based on a two-way mixed-effects model with absolute agreement.

Exploratory analyses and reliability procedures were conducted using IBM SPSS Statistics and Jamovi statistical software, confirmatory factor analysis was performed using IBM SPSS AMOS, and correlation analyses were conducted using Jamovi statistical software (36, 37). Statistical significance was set at *p* < 0.05.

## 3 Results

### 3.1 Characteristics of participants

Sociodemographic, lifestyle, and clinical characteristics of the participants included in the exploratory (EFA) and confirmatory (CFA) samples are presented in Table 1. No statistically significant differences were observed between the two samples across any of the examined variables, indicating a high degree of comparability.

**Table 1:** Sociodemographic, lifestyle, and clinical characteristics of the EFA and CFA samples.

Variable	EFA	CFA	
	*N* (%)	*N* (%)	*p* value
Sex (*n*) *n* (%)			0.532
Male	81 (29.8)	83 (32.3)	
Female	191(70.2)	174 (67.7)	
Marital status *n* (%)			0.542
Single	122 (44.9)	100 (38.9)	
In a relationship/married	140 (51.5)	147 (57.2)	
Divorced	6 (2.2)	7 (2.7)	
Widowed	4 (1.5)	3(1.2)	
Socioeconomic level *n* (%)			0.564
Low	27 (9.9)	33 (12.8)	
Medium	238 (87.5)	217 (84.4)	
High	7 (2.6)	7 (2.7)	
Occupational status *n* (%)			0.101
Student	113 (41.5)	114 (44.4)	
Employed	131 (48.2)	116 (45.1)	
Unemployed	5 (1.8)	13 (5.1)	
Retired	23 (8.5)	14 (5.4)	
Dietary patterns *n* (%)			0.087
Mediterranean	218 (80.1)	198 (77.0)	
Vegetarian	0 (0.0)	3 (1.2)	
Vegan	0 (0.0)	2 (0.8)	
High protein	3 (1.1)	6 (2.3)	
Ketogenic	0 (0.0)	2 (0.8)	
No specific diet	50 (18.4)	42 (16.3)	
Other	1 (0.4)	4 (1.6)	
Family history of eating disorders *n* (%)			0.316
No	254 (93.4)	234 (91.1)	
Yes	18 (6.6)	23 (8.9)	
Family history of obesity *n* (%)			0.356
No	266 (97.8)	254 (98.8)	
Yes	6 (2.2)	3 (1.2)	
Family history of anorexia *n* (%)			0.158
No	271 (99.6)	253 (98.4)	
Yes	1 (0.4)	4 (1.6)	
Family history of bulimia *n* (%)			0.289
No	271 (99.6)	254 (98.8)	
Yes	1 (0.4)	3 (1.2)	
Family history of binge eating *n* (%)			0.596
No	270 (99.3)	256 (99.6)	
Yes	2 (0.7)	1 (0.4)	
Family history of unspecified ED *n* (%)			0.145
No	272 (100.0)	255 (99.2)	
Yes	0 (0.0)	2 (0.8)	
Family history of food-related anxiety *n* (%)			0.091
No	269 (98.9)	257 (100.0)	
Yes	3 (1.1)	0 (0.0)	
Age, mean (SD)	35.3 (16.72)	33.7 (15.45)	0.246
BMI, mean(SD)	24.0 (4.34)	25.3 (4.42)	0.224
Time walking/cycling (minutes), mean (SD)	60.0 (52.99)	59.8 (54.02)	0.978
ONI_total score, mean (SD)	34.4 (8.75)	35.3 (10.04)	0.269
EAT-26 total score, mean (SD)	42.2 (11.51)	43.9 (13.56)	0.128

Both samples were predominantly composed of women (EFA: 70.2%; CFA: 67.7%), with a mean age of 35.3 years (SD = 16.72) in the EFA sample and 33.7 years (SD = 15.45) in the CFA sample. Most participants reported being single or in a stable relationship and belonged to a medium socioeconomic level in both samples.

Regarding occupational status, approximately half of the participants were employed (EFA: 48.2%; CFA: 45.1%), while a substantial proportion were students (EFA: 41.5%; CFA: 44.4%). The Mediterranean diet was the most frequently reported dietary pattern in both samples (EFA: 80.1%; CFA: 77.0%), followed by participants who reported not following any specific diet (EFA: 18.4%; CFA: 16.3%). Vegetarian, vegan, ketogenic, and hyperproteic diets were reported by a small minority of participants in both groups.

Family history of eating-related problems, including eating disorders, obesity, or anxiety related to food, was infrequent in both samples, with prevalence rates below 10% across all categories. Mean body mass index (BMI) values were within the normal-to-overweight range (EFA: 24.0 ± 4.34; CFA: 25.3 ± 4.42), and no significant differences were observed between groups. Similarly, levels of physical activity, measured as time spent walking or cycling, were comparable between samples (EFA: 60.0 ± 52.99 min; CFA: 59.8 ± 54.02 min).

Finally, no significant differences were found between the EFA and CFA samples in total ONI scores (EFA: 34.4 ± 8.75; CFA: 35.3 ± 10.04) or EAT total scores (EFA: 42.2 ± 11.51; CFA: 43.9 ± 13.56), supporting the equivalence of both samples for the purposes of psychometric validation.

### 3.2 Content validity

The professional characteristics of the experts included in the content validity assessment are presented in Table 2. Overall, the expert panel showed a satisfactory level of agreement regarding the relevance and pertinence of the items included in the ONI-ES. At the item level, content validity was assessed using Aiken’s V coefficient. All items achieved Aiken’s V values above the minimum acceptable threshold, indicating adequate content validity according to expert evaluation. Aiken’s V coefficients ranged from 0.71 to 0.96, reflecting a high degree of agreement among experts. Item-level scores assigned by each expert, together with the corresponding Aiken’s V values and their 95% confidence intervals, are presented in Table 2.

**Table 2:** Expert ratings and Aiken’s *V* coefficients for content validity.

Item	Exp 1	Exp 2	Exp 3	Exp 4	Exp 5	Exp 6	Exp 7	Exp 8	Exp 9	Exp 10	Exp 11	Exp 12	Exp 13	Exp 14	Exp 15	Exp 16	Aiken’s *V*	95% CI for Aiken’s *V*
Item 1	4	3	4	4	4	4	4	4	4	4	4	4	4	4	3	4	0.96	0.96 [0.86–0.99]
Item 2	3	3	3	4	3	3	3	4	3	4	3	3	4	4	3	3	0.77	0.77 [0.63–0.88]
Item 3	4	4	3	4	3	4	3	4	3	3	3	4	3	4	4	4	0.85	0.85 [0.63–0.88]
Item 4	4	2	4	4	4	3	4	4	4	4	4	4	4	4	4	4	0.94	0.94 [0.72–0.94]
Item 5	3	2	3	4	4	3	3	4	4	4	3	3	2	3	2	4	0.73	0.73 [0.63–0.88]
Item 6	4	3	3	3	4	3	3	3	3	3	2	3	3	4	3	3	0.71	0.71 [0.58–0.85]
Item 7	3	3	3	4	4	3	4	4	4	4	4	4	4	3	3	4	0.88	0.88 [0.75–0.95]
Item 8	4	3	3	4	4	3	4	4	4	4	4	3	3	4	4	4	0.90	0.90 [0.77–0.97]
Item 9	4	4	4	4	3	4	4	4	4	4	3	4	4	4	4	4	0.96	0.96 [0.86–0.99]
Item 10	4	3	3	4	4	3	4	4	4	4	3	4	2	3	3	4	0.83	0.83 [0.70–0.93]
Item 11	4	3	3	4	4	3	3	4	4	3	4	3	3	2	3	4	0.79	0.79 [0.65–0.90]
Item 12	3	3	3	4	4	3	3	3	4	4	4	4	3	3	4	4	0.83	0.83 [0.70–0.93]
Item 13	4	2	4	4	3	3	4	4	4	4	3	4	4	3	4	4	0.88	0.88 [0.75–0.95]
Item 14	4	2	3	4	4	3	3	3	3	4	2	3	3	2	4	4	0.73	0.73 [0.58–0.85]
Item 15	4	4	4	4	4	3	3	4	3	3	3	3	4	3	3	4	0.83	0.83 [0.70–0.93]
Item 16	4	4	3	4	4	3	4	4	3	4	4	4	3	4	4	4	0.92	0.92 [0.80–0.98]
Item 17	4	3	3	4	4	3	4	4	3	3	4	4	4	4	4	4	0.90	0.90 [0.77–0.97]
Item 18	4	3	3	4	4	3	3	3	3	4	4	3	4	3	3	4	0.81	0.81 [0.67–0.91]
Item 19	2	3	3	4	3	3	3	3	4	4	3	3	2	4	2	4	0.71	0.71 [0.56–0.83]
Item 20	2	3	4	4	3	3	3	4	4	4	4	3	3	2	3	4	0.77	0.77 [0.63–0.88]
Item 21	4	3	4	4	4	4	4	4	4	4	4	4	4	3	4	4	0.96	0.96 [0.86–0.99]
Item 22	4	3	3	4	4	3	4	4	3	4	3	4	4	3	4	4	0.88	0.88 [0.75–0.95]
Item 23	4	3	4	4	4	4	4	4	3	4	4	4	4	3	4	4	0.94	0.94 [0.83–0.99]
Item 24	4	3	3	4	4	4	4	4	4	4	2	3	3	3	4	4	0.85	0.85 [0.72–0.94]

Interpretation suggestion: *V* < 0.70 = questionable; 0.70–0.79 = acceptable; 0.80–0.89 = good; ≥0.90 = excellent.

At the scale level, the overall content validity was considered satisfactory. Based on the quantitative indices and the qualitative feedback provided by the expert panel, no items were eliminated. Minor wording adjustments were implemented in a small number of items to improve clarity and semantic precision, while preserving their original conceptual meaning.

### 3.3 Psychometric properties

#### 3.3.1 Construct validity: exploratory factor analysis (EFA)

Using the first sample (n = 272), the suitability of the data for factor analysis was examined. The Kaiser–Meyer–Olkin (KMO) measure of sampling adequacy was 0.930, indicating excellent suitability for factor analysis. Bartlett’s test of sphericity was statistically significant (*χ*^2^(276) = 5,083, *p* < 0.001), confirming that the correlation matrix was appropriate for factor extraction.

An exploratory factor analysis (EFA) was conducted using Principal Axis Factoring (PAF) with oblimin rotation, allowing correlations among factors. The number of factors retained was determined based on eigenvalues greater than 1, inspection of the scree plot, and parallel analysis. The parallel analysis supported the retention of three factors.

The three-factor solution accounted for 41.9% of the total variance and demonstrated a clear and interpretable factorial structure consistent with the theoretical dimensions of orthorexia nervosa. Item ONI-3 showed factor loadings below 0.30 and high uniqueness and was therefore removed from the final structure. The remaining items demonstrated satisfactory factor loadings and conceptual coherence across the three dimensions.

The three factors were interpreted as follows: (1) orthorexic behaviors and dietary control, (2) emotional attitudes toward food, and (3) social and functional impairment. Although some items presented secondary loadings on other factors, these were lower than the primary loadings and did not compromise the interpretability of the factorial structure. The rotated factor loadings and explained variance are presented in Table 3.

**Table 3:** Rotated factor loadings from the exploratory factor analysis of the ONI-ES.

Item	Components			
	1	2	3	Cronbach’s *α* if item deleted
ONI 6	0.679			0.909
ONI 15	0.671			0.907
ONI 2	0.622			0.909
ONI 17	0.547			0.906
ONI 18	0.544			0.905
ONI 22	0.518			0.904
ONI 8	0.464			0.906
ONI 4	0.460		0.321	0.909
ONI 11	0.456		0.385	0.905
ONI 13		0.849		0.905
ONI 9		0.831		0.905
ONI 21		0.772		0.903
ONI 1		0.581		0.908
ONI 23		0.505		0.903
ONI 7		0.339		0.905
ONI 19			0.678	0.908
ONI 10			0.586	0.909
ONI 5			0.536	0.908
ONI 16			0.519	0.908
ONI 12			0.390	0.909
ONI 24			0.383	0.907
ONI 20			0.374	0.905
ONI 14			0.349	0.910

Extraction method: principal axis factoring. Rotation method: Oblimin with Kaiser normalization.

**Figure 1:**
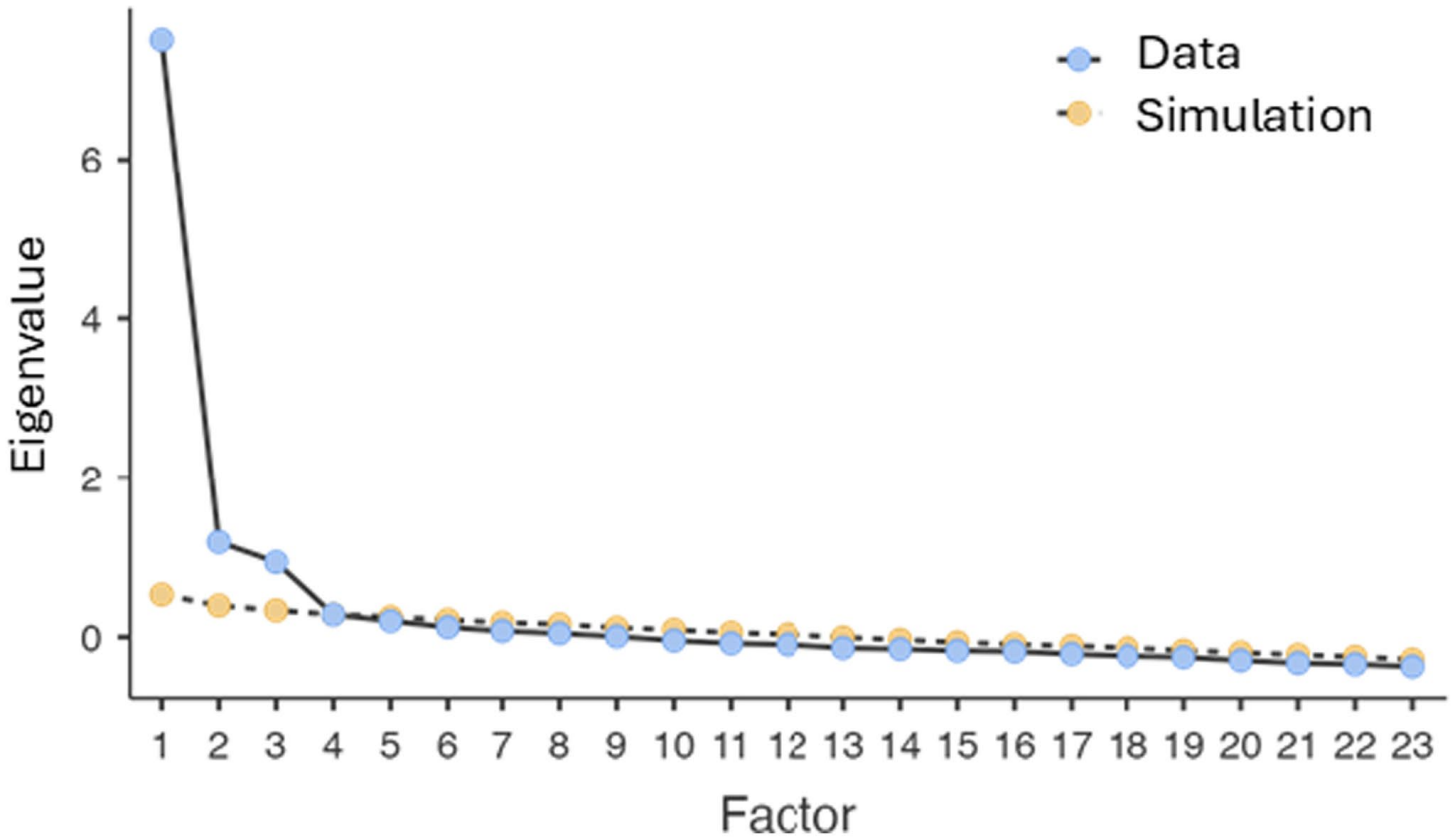
Parallel analysis scree plot showing observed and simulated eigenvalues for factor retention.

Parallel analysis confirmed the adequacy of a three-factor solution, as the first three observed eigenvalues exceeded those obtained from randomly generated data. The parallel analysis provided empirical support beyond the Kaiser criterion see Figure 1.

#### 3.3.2 Structural confirmation: confirmatory factor analysis (CFA)

The three-factor model identified in the exploratory phase was subsequently tested through confirmatory factor analysis (CFA) using an independent sample (*n* = 257). The CFA supported the previously identified factorial structure, confirming the adequacy of the three-dimensional model comprising orthorexic behaviors, emotional attitudes toward food, and social and functional impairment.

Model fit indices indicated an acceptable overall fit to the data, with a Comparative Fit Index (CFI) of 0.905, a Tucker–Lewis Index (TLI) of 0.891, and a Root Mean Square Error of Approximation (RMSEA) of 0.066 (90% confidence interval [CI] = 0.057–0.074). These values are consistent with commonly recommended thresholds for acceptable model fit in psychometric validation studies.

**Figure 2:**
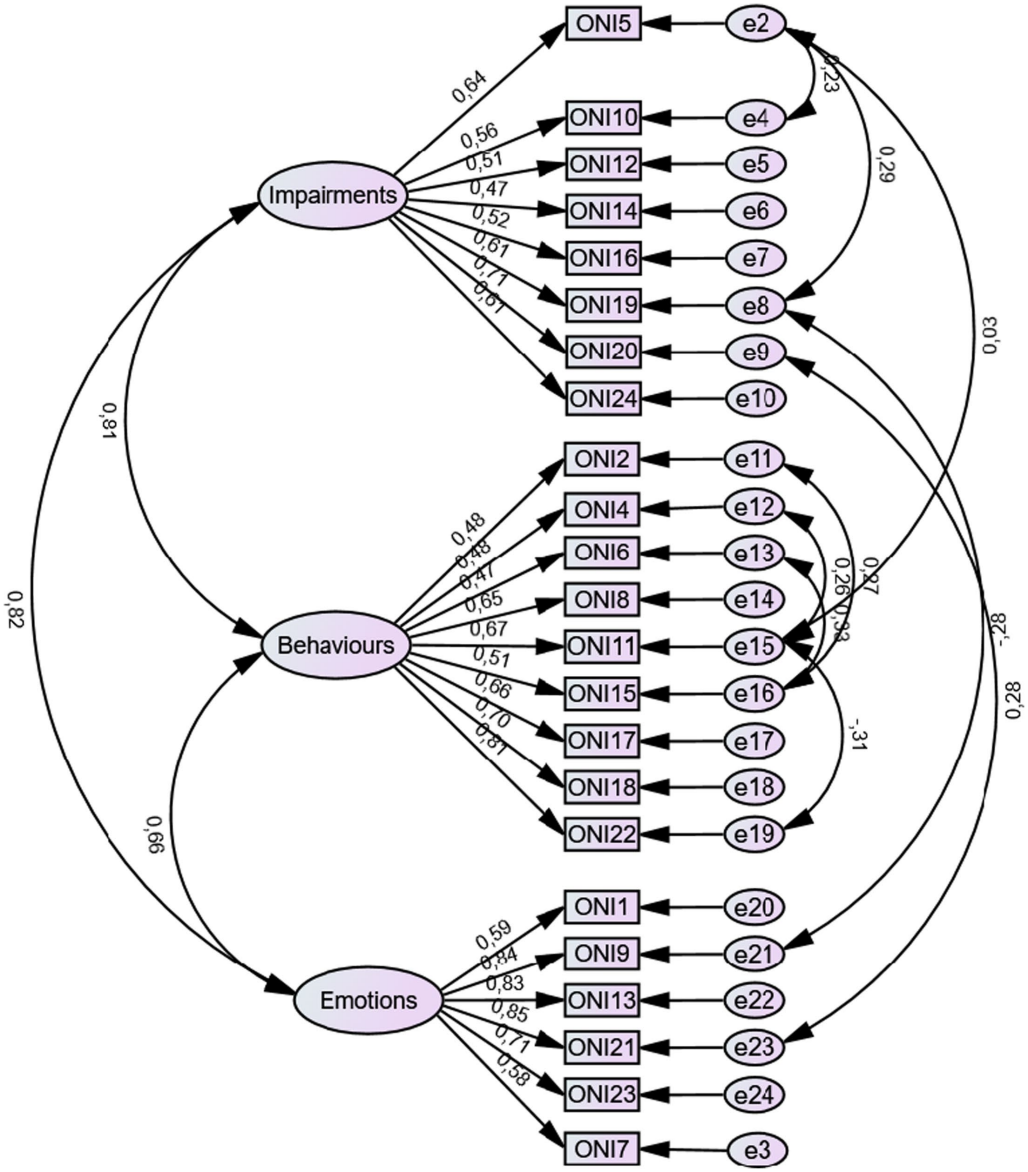
Confirmatory factor analysis of the three-factor model of the ONI-ES.

The standardized factor loadings and correlations between latent factors are presented in Figure 2.

#### 3.3.3 Convergent validity

A positive correlation of moderate-to-high magnitude was observed between ONI-ES total scores and EAT-26 total scores (*r* = 0.586, *df* = 519, *N* = 521, *p* < 0.001) supporting the convergent validity of the instrument while indicating that orthorexic tendencies overlap with, but are not reducible to, general disordered eating attitudes. Due to missing data in the EAT-26 responses, eight participants were excluded from this analysis, resulting in a reduced sample size (*n* = 521).

### 3.4 Reliability

#### 3.4.1 Internal consistency (*α* and *ω*)

The internal consistency of the Spanish version of the Orthorexia Nervosa Inventory (ONI-ES) was evaluated using Cronbach’s alpha and McDonald’s omega coefficients. The total scale demonstrated excellent internal consistency (*α* = 0.91; *ω* = 0.92). At the subscale level, Cronbach’s alpha values were 0.84 for Orthorexic Behaviors, 0.87 for Emotional factor, and 0.80 for Impairment, while McDonald’s omega coefficients ranged from 0.80 to 0.87, indicating good to excellent reliability. Detailed reliability indices are presented in Table 4.

**Table 4:** Internal consistency indices for the ONI-ES.

Scale/subscale	Cronbach’s *α*	McDonald’s *ω*
ONI-ES Total	0.910	0.915
Behaviors	0.843	0.847
Emotional	0.865	0.873
Impairment	0.798	0.801

#### 3.4.2 Test–retest reliability

Test–retest reliability was assessed using the intraclass correlation coefficient (ICC) based on a two-way mixed-effects model with absolute agreement, in a subsample of 31 participants who completed the questionnaire on two occasions separated by a 15-day interval. The results indicated good to excellent temporal stability. For single measures, the ICC was 0.824 (95% CI: 0.664–0.912; *p* < 0.001), while for average measures the ICC was 0.903 (95% CI: 0.798–0.954; *p* < 0.001).

## 4 Discussion

The present study aimed to culturally adapt and validate the Orthorexia Nervosa Inventory (ONI) for use in a Spanish general population. The findings provide strong support for the psychometric adequacy of the ONI-ES, confirming its three-factor structure and demonstrating excellent internal consistency and temporal stability. Both exploratory and confirmatory analyses supported a model comprising orthorexic behaviors, emotional attitudes toward food, and social and functional impairment. The proportion of explained variance observed in the present study (41.9%) may be considered moderate; however, this finding should be interpreted within the context of multidimensional psychological constructs. Instruments assessing complex phenomena such as eating behavior and health-related beliefs typically yield moderate explained variance due to the heterogeneity and multifaceted nature of these constructs. In addition, the use of Principal Axis Factoring, which focuses on shared variance rather than total variance, may contribute to lower explained variance compared to data-reduction approaches such as principal component analysis (38–40).

Together with the satisfactory fit indices obtained in the confirmatory phase, these findings reinforce the structural robustness of the ONI-ES. Importantly, the exploratory factor analysis was conducted using Principal Axis Factoring with oblimin rotation, allowing correlations among factors and providing a more appropriate estimation of latent constructs. In this revised analysis, item ONI-3 showed low factor loadings and high uniqueness and was therefore removed from the final structure. Additionally, item ONI-7 loaded more strongly on a different factor than in the original instrument and was therefore reassigned based on statistical and conceptual coherence. The remaining items demonstrated satisfactory loadings and conceptual consistency across the three dimensions, supporting the stability of the factor structure.

This replication of the original three-dimensional structure aligns with previous validations conducted in Italian, Turkish, Hungarian, and Chinese samples (26–29), supporting the cross-cultural stability of the instrument. Although differences emerged, including the removal of one item and the reassignment of item 7, the overall factorial organization remained conceptually aligned with the original model. Variations in item allocation across factors have also been observed in previous cross-cultural validations — notably, the Chinese validation reported substantial differences in item distribution, highlighting how orthorexic tendencies may manifest differently depending on cultural and dietary contexts (28). The present study extends these findings to a heterogeneous general adult population, whereas several prior validations were conducted primarily in student or more specific samples. The consistency of the three-dimensional solution across languages, countries, and population types suggests that the dimensions assessed by the ONI reflect stable components of orthorexic symptomatology rather than sample-specific or culturally idiosyncratic patterns.

This structural stability is particularly relevant in light of the broader psychometric challenges that have characterized earlier measures of orthorexia (41–44). Previous instruments, particularly the ORTO-15, have demonstrated variability in factorial solutions and inconsistent reliability estimates across cultural adaptations, which has led to ongoing debate regarding their psychometric adequacy (42, 45, 46). In contrast, the ONI has consistently retained its multidimensional configuration across countries and languages (26–29). The present findings contribute to this growing body of evidence, supporting the ONI as a more psychometrically robust and conceptually coherent instrument for assessing orthorexic tendencies. The moderate correlation observed between ONI-ES and EAT-26 scores further supports the convergent validity of the instrument. Although orthorexic tendencies showed substantial overlap with disordered eating attitudes, the correlation was far from unity, suggesting that orthorexia shares common features with traditional eating disorders while maintaining conceptual distinctiveness. This finding is consistent with previous literature indicating partial but not complete convergence between orthorexia nervosa and established eating disorder symptomatology (1, 15, 47–51). Moreover, it is important to acknowledge that orthorexia nervosa remains a construct without a universally accepted definition. Although the three-factor structure was supported in this study, this finding should not be interpreted as definitive evidence of the construct’s dimensionality. Validation studies may inadvertently reinforce the theoretical assumptions underlying the original instrument, a phenomenon sometimes described as circular validation in psychometric research. Therefore, future studies should continue to critically examine alternative conceptualizations and explore whether different populations or cultural contexts yield distinct structural configurations.

From a theoretical standpoint, the identification of distinct behavioral, emotional–cognitive, and functional dimensions supports the importance of conceptualizing orthorexia as a complex and multidimensional construct. This perspective is consistent with the original development of the Orthorexia Nervosa Inventory, which explicitly incorporated behavioral, affective, and impairment components (25), as well as with subsequent theoretical and empirical work emphasizing the cognitive rigidity and emotional investment associated with orthorexic symptomatology (4, 50). Reducing orthorexic tendencies to dietary practices alone may overlook the psychological involvement and potential impairment that differentiate pathological orthorexia from non-pathological healthy eating behaviors. The present validation supports the view that orthorexia involves not only restrictive behaviors but also cognitive–emotional engagement and functional consequences, aligning with contemporary multidimensional models of maladaptive eating patterns.

The confirmation of differentiated dimensions also contributes to the ongoing debate regarding the nosological status of orthorexia nervosa (52). Although orthorexic tendencies share certain features with established eating disorders—such as dietary rigidity and preoccupation with food—the multidimensional organization observed in the ONI suggests that the construct cannot be reduced to restrictive eating behaviors alone. The presence of a distinct emotional component and a separate functional impairment dimension supports the interpretation of orthorexia as a structured pattern of cognitive involvement and psychosocial impact (24, 53). This structural differentiation may help clarify discussions about whether orthorexia represents a variant of existing eating disorders or a related but distinguishable condition characterized by its focus on food purity and health-oriented restriction (54, 55).

The strong reliability indices observed in the present study reinforce the psychometric robustness of the ONI-ES. Both internal consistency estimates (Cronbach’s alpha and McDonald’s omega) demonstrated good to excellent coherence across the total scale and its subdimensions, while the satisfactory test–retest stability over a 15-day interval suggests that the instrument captures relatively stable orthorexic tendencies rather than transient fluctuations in eating-related attitudes. Together, these findings indicate that the ONI-ES provides a consistent and temporally stable assessment framework, comparable to reliability levels reported in previous cross-cultural validations of the instrument.

From a practical perspective, the availability of a psychometrically sound Spanish version of the ONI provides researchers and clinicians with a comprehensive tool for assessing orthorexic tendencies in Spanish-speaking populations. Its multidimensional nature allows for nuanced evaluation of symptom presentation, facilitating research applications, epidemiological studies, and potential early identification in community contexts.

Several limitations should be acknowledged. First, the use of a non-probabilistic convenience sample and online recruitment strategy may limit the generalizability of the findings. Additionally, the sample was predominantly composed of women and relatively young participants, which may further restrict the applicability of the results to more diverse or clinical populations. Second, the cross-sectional design precludes conclusions regarding symptom development over time. Longitudinal studies are needed to better understand the stability and evolution of orthorexic tendencies. Third, the study relied exclusively on self-report instruments, which may introduce response bias, including social desirability and subjective interpretation of items. Finally, although convergent validity was assessed using the EAT-26, additional psychological constructs such as anxiety, obsessive-compulsive traits, and perfectionism were not included, which limits the assessment of discriminant validity. Future research should incorporate these measures to more comprehensively examine the distinctiveness of orthorexia and better clarify its position within the broader nomological network of related psychological constructs.

## 5 Conclusion

In conclusion, the ONI-ES demonstrates solid psychometric properties and supports a three-factor structure in a Spanish general population. Despite minor modifications, including the removal of one item and the reassignment of another, the instrument showed good reliability, structural validity, and cross-cultural applicability as a multidimensional measure of orthorexic symptomatology. These findings support the use of the ONI-ES in research and clinical contexts within Spanish-speaking populations.

## Data Availability

The raw data supporting the conclusions of this article will be made available by the authors, without undue reservation.
